# Antibiotic resistance of *Helicobacter pylori* isolated from patients in Nanjing, China: A cross-section study from 2018 to 2021

**DOI:** 10.3389/fcimb.2022.970630

**Published:** 2022-09-08

**Authors:** Zongdan Jiang, Xuetian Qian, Zhi Wang, Yunfan Dong, Yuqin Pan, Zhenyu Zhang, Shukui Wang

**Affiliations:** ^1^ Department of Gastroenterology, Nanjing First Hospital, Nanjing Medical University, Nanjing, China; ^2^ General Clinical Research Center, Nanjing First Hospital, Nanjing Medical University, Nanjing, China

**Keywords:** *Helicobacter pylori*, antibiotic resistance, ARMS-PCR, Nanjing, clarithromycin

## Abstract

The increasing antibiotic resistance of *Helicobacter pylori* infection is a globally urging problem. To investigate the *H. pylori* resistance situation in Nanjing, China, we enrolled patients in Nanjing First Hospital from January 2018 to May 2021. *H. pylori* strains were isolated from patients who had at least one positive 13C-urea breath or rapid urease result. Subsequently, we performed antibiotic susceptibility tests on the isolated strains to clarithromycin, metronidazole, levofloxacin, amoxicillin, furazolidone and tetracycline. ARMS-PCR was conducted to determine *H. pylori* clarithromycin resistance gene mutation. Our results demonstrated that the primary resistance rates of metronidazole, clarithromycin, levofloxacin, amoxicillin, furazolidone and tetracycline were 67.19% (1417/2109), 35.99% (759/2109), 24.23% (511/2109), 0.76% (16/2109), 0.28% (6/2109) and 0.09% (2/2109), respectively. The resistance rates of metronidazole, clarithromycin and levofloxacin elevated significantly after treatment and the three antibiotics composed the majority of multi-resistance patterns. However, the resistance rates of amoxicillin, furazolidone and tetracycline were still in low levels after treatment. ARMS-PCR showed a rather good consistency with antibiotic susceptibility test in detecting clarithromycin resistance, with a kappa value of 0.79. Overall, this study revealed the latest complex situation of antibiotic resistance of *H. pylori* infection in Nanjing and offered suggestions on clinical medication for curing *H. pylori*.

## Introduction

*Helicobacter pylori*, a transmissible pathogen, colonizes in the stomach of around a half of the world population (Hooi et al., 2017). *H. pylori* infection causes gastritis and peptic ulcers and may lead to stomach cancer and gastric mucosa associated lymphoid tissue lymphoma. It has been listed as a carcinogen worldwide (NIH Consensus Conference, 1994). Thus, eradication of *H. pylori* infection is of great value for improving public health.

However, curing *H. pylori* infection still remains a challenge. Only a few antibiotics, namely clarithromycin, amoxicillin, metronidazole, levofloxacin, tetracycline, furazolidone and rifabutin, are available for effectively eradicating *H. pylori* (Malfertheiner et al., 2017; Zhuge et al., 2018). The extensive use of the limited antibiotics leads to the urging problem of rapid increasing antibiotic resistance. Most *H. pylori* resistance is caused by mutations encoding chromosomal changes that alter the drug target or inhibit the effectiveness (Gong and Yuan, 2018). Alternatively, physiological changes and cellular adaptations may also contribute to drug resistance in *H. pylori*, including biofilms and coccoid formation, as well as altered regulation of drug uptake and efflux (Tshibangu-Kabamba and Yamaoka, 2021). The resistance of *H. pylori* leads to a series of tackles, such as failures in treatment, diagnostic challenges and clinically ambiguous therapeutic outcomes. China has a large *H. pylori* infected population that exhibits complicated antibiotic resistance features (Xie and Lu, 2015). The antibiotic resistance rates differed among regions. For example, the resistance rate of clarithromycin varied from 20% to 50% in different areas of China (Hu et al., 2017; Liu et al., 2018). Thus, investigating the local antibiotics resistance patterns would be most beneficial for guiding clinical medication of *H. pylori*. However, the data in Nanjing, China was limited.

The aim of the study was to explore the present situation of antibiotics resistance in Nanjing and offer advices on clinical medication of *H. pylori*. We isolated *H. pylori* strains from patients who had at least one positive 13C-urea breath or rapid urease result and subsequently performed antibiotic susceptibility tests to clarithromycin, metronidazole, levofloxacin, amoxicillin, furazolidone and tetracycline. We also compared the consistency between the antibiotic susceptibility results and the amplification refractory mutation system combined with quantitative real‐time PCR (ARMS‐PCR) results for detecting clarithromycin resistance.

## Materials and methods

### Patients and *H. pylori* strains

**Figure 1:**
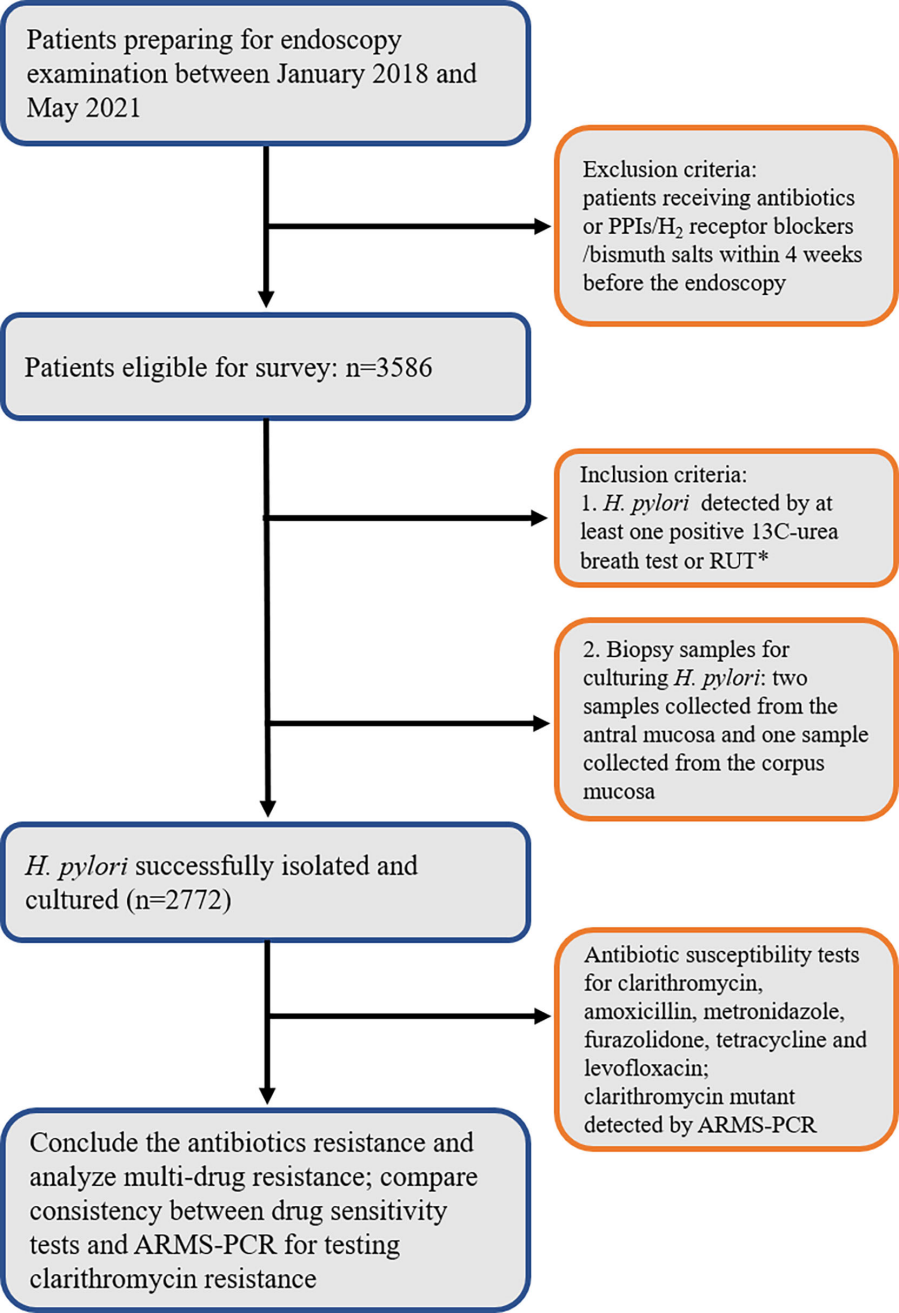
Flowchart depicting the study design. * RUT, rapid urease test.

Patients preparing for endoscopy examination were recruited in Nanjing First Hospital from January 2018 to May 2021. The inclusion criteria were as follows. (i) Participants had at least one positive 13C-urea breath or rapid urease result for *H. pylori*. (ii) Gastric mucosa specimens were obtained from the participants who gave informed consent. Two specimens were taken from the antral mucosa and one specimen was taken from the antral body during endoscopy for culturing *H. pylori* next. Participants receiving antibiotics, proton-pump inhibitors (PPIs), H_2_ receptor blockers or bismuth salts within 4 weeks before the endoscopy were excluded. Finally, a total of 2772 patients were enrolled for the study (**Figure 1**). The study was reviewed and approved by the Ethics Committee of Nanjing First Hospital.

### *H. pylori* culture

We restored the isolation culture tube to 25~37°C and put the mucosal biopsy specimens into the same tube. Then we cultured them in a 37°C incubator for 3~7 days and observed the colony morphology and separation effect in the separation culture tube every day. When the culture medium turned red and the particles increased, we identified and amplified the strains. We used the conventional Gram staining to assist the identification. Under the microscope, typical *H. pylori* is gram negative, red and diverse in morphology, mainly in gull shape, spiral shape, s shape or long arc shape.

### Antibiotic susceptibility test

We scraped an appropriate amount of *H. pylori* colonies without pollution, with good colony morphology and typical microscopic morphology after 72 hours of pure culture with sterile cotton swabs in 0.45% NaCl sterilization solution, and adjusted the concentration of bacterial suspension to 2.0 Michaelis units (6×10^8^ CFU/mL). Then 100μL bacteria suspension was evenly coated on MH blood agar culture medium, and E test strips of clarithromycin, metronidazole, levofloxacin, amoxicillin, furazolidone and tetracycline were pasted, respectively. It was cultured for 72 hours in a micro aerobic environment with 35°C and humidity greater than 95%. The minimum inhibitory concentration (MIC) of *H. pylori* to the drug was read at the intersection of the inhibition circle and E test strip.

The resistance breakpoints of antibiotics were referred to previous reports (Megraud et al., 1999; Megraud and Lehours, 2007) or the Clinical and Laboratory Standards Institute (CLSI) guidelines (Zhang et al., 2015). Clarithromycin≥1mg/l, metronidazole≥8mg/l, levofloxacin≥2mg/l, amoxicillin≥0.5mg/l, furazolidone≥2 mg/l and tetracycline≥1mg/l were determined as drug resistance. *H. pylori* strain ATCC43504 was included as an antibiotic susceptibility testing quality control. Antibiotic susceptibility tests were conducted at the Institute of Gastroenterology, Nanjing First Hospital, Nanjing Medical University.

### DNA extraction

The HiPure Bacterial DNA Kit (D3146-03, Guangzhou Magen Biotechnology Co.) was used for DNA extraction. Briefly, after the bacteria were collected by centrifugation, the cell wall was removed by lysozyme digestion. DNA was released from the cell after adding the lysate and proteinase K. Then the binding solution was added to adjust the optimal binding conditions. Next, the solution was transferred to the purification column and centrifuged. DNA was bound to the filter membrane, and impurities such as proteins were filtered out into the filtrate. Residual contaminants and enzyme inhibitors were removed after two washing steps, and the DNA was finally eluted with a small amount of buffer.

### Detection by ARMS-PCR

The major cause of clarithromycin resistance in *H. pylori* strains is point mutations in domain V of the 23S ribosomal RNA gene (A2142G, A2143G), which decrease the affinity of the ribosome for the drug and, therefore, the bacteria become resistant. Thus, we can detect the mutations by ARMS-PCR to assist determine the clarithromycin resistance. The PCR system was composed of 10 × PCR buffer, 2 mM MgCl_2_, 0.2 mM dNTPs, 2U Taq enzyme, 0.3 µM common reverse primer, and 0.1 µM common reverse probe. The sample was divided into 3 tubes; then, 3 forward primers were added. Finally, the DNA template was added. The reaction conditions were as follows: pre-denaturation at 94°C for 5 min, denaturation at 94°C for 15 s, and extension at 62°C for 30 s for 40 amplification cycles. Fluorescence signals were collected for each annealing temperature of each cycle. The primers and probe were listed in the **Supplementary Table 1**.

### Sequencing verification

We used the PCR sequencing for verifying the accuracy of the ARMS‐PCR detection results. The PrimeSTAR^®^ HS (Premix) (Dalian TaKaRa Co., Ltd.) was used. The reaction system contained 20 µL PrimeSTAR HS, 10 pmol forward primer, 10 pmol reverse primer and 100 ng DNA template. The reaction conditions were as follows: pre-denaturation at 94°C for 15 s, degeneration at 98°C for 10 s, extension at 55°C for 15 s, and extension at 72°C extension for 1 min for 30 amplification cycles. Subsequently, the PCR products were purified, and then a BigDye Terminator v3.1 Cycle Sequencing Kit (QIAGEN) and a 3730 DNA Analyzer (Applied Biosystems, Life Technologies) were used for sequencing and analyzing the DNA products respectively. The forward primer was 5’-AATGGCGTAACGAGATGGGAG-3’ and the reverse primer was 5’-TCCATAAGAGCCAAAGCCCTTAC-3’.

### Statistical analysis

Data analysis was performed by SPSS 26.0 (IBM SPSS, Chicago, IL, USA). Percentages were used to describe the antibiotic resistance rates of *H. pylori*. The sensitivity and the specificity of ARMS‐PCR for detecting *H. pylori* clarithromycin resistance was calculated by Chi-square test. Kappa was calculated to estimate concordance between ARMS‐PCR and antibiotic susceptibility testing. A kappa value of > 0.75 indicated a high consistency.

## Results

### Antibiotic resistance rates of *H. pylori*

According to the antibiotic treatment times, we divided the enrolled 2772 patients into three groups, namely first-time treatment or before treatment (n=2109), second-time treatment (n=261) and treatment over 2 times (n=402). As shown in **Table 1**, in Nanjing area, up to 67.19% (1417/2109) patients was metronidazole resistant for the first-time treatment of *H. pylori*. The resistance rates of metronidazole increased to 71.26% (186/261) (second-time treatment) and 81.84% (329/402) (treatment over 2 times). Obvious upward trends were also seen in clarithromycin resistance and levofloxacin resistance with increasing treatment numbers. Approximately one-third and one-fourth of patients was clarithromycin resistant and levofloxacin resistant before treatment, respectively. After more than 2 times of treatment, both of clarithromycin and levofloxacin resistance rates were greater than 50%.

**Table 1:** Antibiotic resistance rates of *H. pylori* before treatment or after different times of treatment in Nanjing, China.

Resistance rate (%)	Metronidazole	Clarithromycin	Levofloxacin	Amoxicillin	Furazolidone	Tetracycline
**before treatment**	67.19 (1417/2109)	35.99 (759/2109)	24.23 (511/2109)	0.76 (16/2109)	0.28 (6/2109)	0.09 (2/2109)
**for the second-time treatment**	71.26 (186/261)	69.73 (182/261)	42.15 (110/261)	1.15 (3/261)	0.38 (1/261)	0 (0/261)
**for treatment more than 2 times**	81.84 (329/402)	83.83 (337/402)	69.40 (279/402)	1.49 (6/402)	0.25 (1/402)	0 (0/402)

Unlike the above three high-level resistant antibiotics, amoxicillin, furazolidone and tetracycline were shown as low-level resistant. Only a tiny increase was observed in amoxicillin resistance rates, i.e., 0.76% (16/2109), 1.15 (3/261) and 1.49% (6/402). On the other hand, the resistance rates of furazolidone and tetracycline were consistently low, less than 0.5%.

### Multiple antibiotics resistance of *H. pylori*

**Figure 2:**
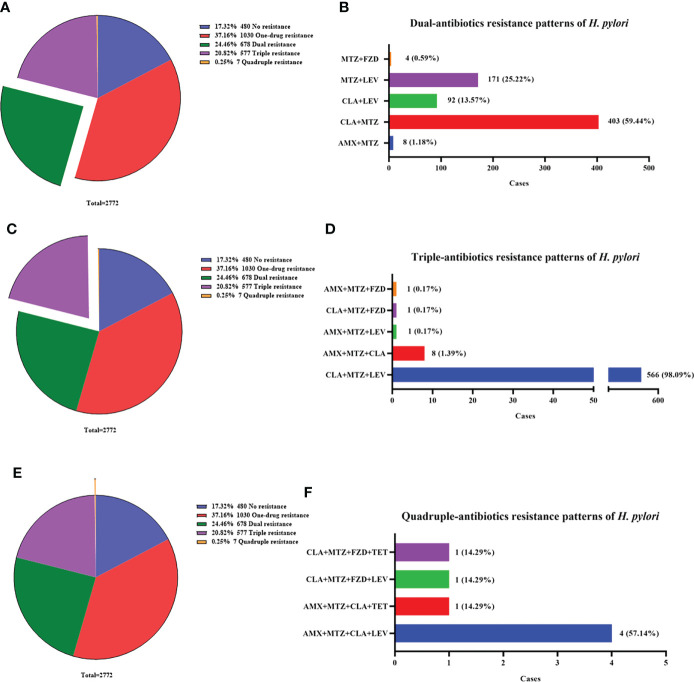
Multi-antibiotics resistance patterns of *H pylori*. **(A, B)** Dual-antibiotics resistance patterns. **(A)** Composition of antibiotics resistance patterns. The green part represented the proportion of total dual-antibiotics resistance patterns, accounting for 24.46% (678/2772). **(B)** Composition of dual-antibiotics resistance patterns. **(C, D)** Triple-antibiotics resistance patterns. **(C)** Composition of antibiotics resistance patterns. The purple part represented the proportion of total triple-antibiotics resistance patterns, accounting for 20.82% (577/2772). **(D)** Composition of triple-antibiotics resistance patterns. **(E, F)** Quadruple-antibiotics resistance patterns. **(E)** Composition of antibiotics resistance patterns. The orange part represented the proportion of total quadruple-antibiotics resistance patterns, accounting for 0.25% (7/2772). **(F)** Composition of quadruple-antibiotics resistance patterns. AMX, amoxicillin; CLA, clarithromycin; FZD, furazolidone; MTZ, metronidazole; LEV, levofloxacin; TET, tetracycline.

To gain a better insight into the antibiotic resistance situation of *H. pylori* in Nanjing, we further analyzed the resistance patterns, including no resistance (17.32%, 480/2772), one-drug resistance (37.16%, 1030/2772), dual resistance (24.46%, 678/2772), triple resistance (20.82%, 577/2772) and quadruple resistance (0.25%, 7/2772) (**Figures 2A, C, E**).

The most common dual resistance pattern was clarithromycin plus metronidazole resistance (59.44%, 403/678) (**Figure 2B**). The other patterns were composed of metronidazole plus levofloxacin resistance (25.22%, 171/678), clarithromycin plus levofloxacin resistance (13.57%, 92/678), amoxicillin plus metronidazole resistance (1.18%, 8/678) and metronidazole plus furazolidone resistance (0.59%, 4/678) (**Figure 2B**).

Among triple resistance patterns, the combination of clarithromycin, metronidazole and levofloxacin accounted for the most up to 98.09% (566/577). The other patterns were few and listed in **Figure 2D**.

Quadruple resistance patterns were rare but still existed, with a low proportion of 0.25% (7/2772). The patterns included amoxicillin, metronidazole, clarithromycin plus levofloxacin resistance (57.14%, 4/7), amoxicillin, metronidazole, clarithromycin plus tetracycline resistance (14.29%, 1/7), metronidazole, clarithromycin, levofloxacin plus furazolidone resistance (14.29%, 1/7) and metronidazole, clarithromycin, tetracycline plus furazolidone resistance (14.29%, 1/7) (**Figure 2F**).

### Comparison of the antibiotic susceptibility test and ARMS-PCR detection of *H. pylori* clarithromycin resistance gene mutation

Clarithromycin is recommended in the first-line therapy for *H. pylori* eradication among guidelines, with the advantage of few side effects. However, based on our data, the clarithromycin resistance rate in Nanjing area was too high to consider it as the first-line antibiotic. In this clinical dilemma, ARMS-PCR, a simple and rapid method for detecting *H. pylori* clarithromycin resistance 23S rRNA mutation, seemed to be of benefit for guiding precision antibiotics utilization.

A total of 2723 samples were tested by ARMS-PCR. There were 1439 mutations detected, including A2143G homozygous mutation (n=1190), A2143G heterozygous mutation (n=246), A2142G homozygous mutation (n=2) and A2142G heterozygous mutation plus A2143G heterozygous mutation (n=1) (**Table 2**). Compared with the gold standard of drug sensitivity test, the sensitivity and specificity of ARMS-PCR were 96.23% and 83.86%, respectively (**Table 3**). In addition, ARMS-PCR also displayed a high consistency with drug sensitivity result, with a kappa value of 0.79 (*P*<0.001) (**Table 4**).

**Table 2:** Cases of different mutation types in 23S rRNA of *H. pylori*.

	Mutation type	Cases
	A2142G homozygous mutation	2
	A2142G heterozygous mutation plus A2143G heterozygous mutation	1
	A2143G homozygous mutation	1190
	A2143G heterozygous mutation	246
**Total**		1439

**Table 3:** Comparison of the antibiotic susceptibility test and ARMS-fluorescence PCR detection of *H. pylori* clarithromycin resistance gene mutation.

		Antibiotic susceptibility test (gold standard)		Total
		Resistance phenotype	Sensitive phenotype	
**ARMS‐fluorescence PCR test result**	**Mutation**	1201	238	1439
	**Wild**	47	1237	1284
	**Total**	1248	1475	2723
		Sensitivity: 96.23%	Specificity: 83. 86%	

**Table 4:** Consistency evaluation of two methods for *H. pylori* resistance (n = 2723).

	Value	Asymp. Std. Error	Approx. Tb	Approx. Sig.
**Kappa**	0.79	0.01	41.72	<0.001

## Discussion

*H. pylori* is an important human pathogen which plays a significant role in the pathogenesis of upper gastrointestinal tract diseases. In developed countries, the infection rates of *H. pylori* range from 25% to 50%, whereas in developing countries, the rate is up to 80%. The infection rate in China is also numbered as high as 56.2% (Alba et al., 2017; Dolan et al., 2018). The increasing antibiotics resistance of *H. pylori* has been a great concern globally (Song et al., 2014; Alba et al., 2017). Over different countries and regions, metronidazole resistance rates vary from 1% to 83% (Karpinski et al., 2015; Ang et al., 2016; Gunnarsdottir et al., 2017). Although resistance rates of clarithromycin are not as high as those of metronidazole in most countries, the values are still vexing, most over 20% (Goudarzi et al., 2016; Alarcon et al., 2017). Nanjing is a city with a population of more than 10 million. However, few studies about the antibiotics resistance of *H. pylori* have been conducted here (Jiang et al., 2021). Our center continues to focus on the issue and try to give our suggestions on clinical medication of *H. pylori* in Nanjing.

Our previous study reported that in Nanjing the resistance rates of metronidazole, clarithromycin, levofloxacin, amoxicillin, furazolidone and tetracycline before treatment were 78.57%, 38.62%, 27.41%, 1.83%, 0.58% and 0.33%, respectively (Jiang et al., 2021). Throughout the study, the latest resistance rates of metronidazole, clarithromycin, levofloxacin, amoxicillin, furazolidone and tetracycline before treatment were 67.19%, 35.99%, 24.23%, 0.76%, 0.28% and 0.09%, respectively. Gratifyingly, we observed an obvious decrease of primary resistance rate of each antibiotic. However, we still cannot ignore the problem that the resistance rates of metronidazole, clarithromycin and levofloxacin are still in high levels. As shown in **Table 1**, for patients with failure of first treatment, the resistance rates of the above three antibiotics elevated incredibly, further intensifying the complex situation of multiple antibiotics resistance. Thankfully, for these patients, we still can choose amoxicillin, furazolidone and tetracycline in an empiric therapy. Proton pump inhibitor-amoxicillin-containing high dose dual therapy (HDDT) has been proposed as the first-line treatment in an area with high prevalence of antibiotic resistance (Yu et al., 2019; Gao et al., 2020). HDDT was a therapy with both amoxicillin (≥2.0 g/day) and PPIs more than twice daily for 2 weeks. Compared with the current guidelines-recommended therapies, HDDT also achieved similar eradication rates varying from 74.6%~95.7%, with fewer side effects (Miehlke et al., 2006; Sapmaz et al., 2017; Tai et al., 2019). Thus, we suggest that HDDT can be a good choice for the first-line treatment or as a compensatory therapy after a failure eradication in Nanjing.

On the other hand, both furazolidone and tetracycline still keep very low resistance rates in Nanjing, less than 0.5%. They also offer the other choices for patients with multiple treatment failures. However, a higher number of side effects of the two antibiotics limit the use (Sanchez et al., 2004; Zhuge et al., 2018). For example, use of tetracycline and other tetracycline-derivatives may result in permanent discoloration of dentitions. Taken together, it would not be a good choice for us to completely discard the use of metronidazole, clarithromycin and levofloxacin. The primary resistance rates of metronidazole, clarithromycin and levofloxacin were too high to recommend them in first-line empiric treatment. However, it would be convincible if antibiotic susceptibility tests available before therapy-decision making. The high requirements for *H. pylori* culture and slow growth limit the use of antibiotic susceptibility tests in clinic. Thus, we need to find another simple and accurate way to assist us make decisions.

The main molecular mechanism of *H. pylori* resistance to clarithromycin is caused by the point mutation in the V region of 23S rRNA (Stone et al., 1996; Taylor et al., 1997; Vianna et al., 2016). These mutations are able to disrupt the peptidyl transferase loop conformation and inhibit the binding between clarithromycin and the 23S rRNA, reducing its efficiency and leading to a resistance phenotype (Occhialini et al., 1997). ARMS-PCR has been developed as a simple and rapid method for detecting *H. pylori* clarithromycin resistance 23S rRNA mutation. In our study, compared with antibiotic susceptibility tests, ARMS-PCR showed an excellent sensitivity of 96.23% and a good specificity of 83.86%. Besides, ARMS-PCR also showed a high consistency with antibiotic susceptibility tests, with a kappa value of 0.79. Thus, we suggest ARMS-PCR can be applied in clinic for guiding the use of clarithromycin.

According to our study, less than one fifth (17.32%) of patients were sensitive to the all six antibiotics. Up to 45.53% patients were resistant to more than one antibiotic. Metronidazole, clarithromycin and levofloxacin formed the majority patterns of multi-antibiotics resistance. Likewise, a multi-region study in China also demonstrated the similar result (Song et al., 2014). This indicates that when one of the three antibiotics has been resistant, it should be tailored carefully to choose the other two for an eradication therapy. No patients in our cohort were found as quintuple or sextuple resistance. Although the quadruple resistance rate was low, its existence was still vexing. There are still no effective solutions for solving the problem of multi-antibiotic resistance. Strengthening management of antibiotic use, improving the level of medical knowledge among physicians in healthcare institutions, and increasing publicity and education about the rational use of antibiotics could be the only choices.

This study has some limitations. First of all, the samples in this study were from a single-center in Nanjing, which could bring bias into the study and could not reflect *H. pylori* patterns in the general population in the area. Second, the mucosal biopsy specimens were collected from the antrum and corpus, and put into the same vial for culturing. We did not conduct culture separately; thus, our results could not reflect the antibiotics resistance profiles of *H. pylori* of each localization in the stomach. This could lead to an underestimate of the antimicrobial resistance rates.

In conclusion, compared with the past years, the antibiotic resistance situation of *H. pylori* infection in Nanjing has been improved, with a reduction of primary resistance rate of each antibiotic. However, the resistance rates of metronidazole, clarithromycin and levofloxacin are still rather high. Antibiotic susceptibility tests are necessary before using any of the three antibiotics in the eradication therapy. ARMS-PCR would be a good choice for detecting clarithromycin resistance. The resistance rates of amoxicillin, furazolidone and tetracycline are still low. They can be recommended in the first-line empiric treatment in Nanjing. Besides, HDDT would be a rather good choice for curing *H. pylori*.

## Data availability statement

The original contributions presented in the study are included in the article/**Supplementary Material**. Further inquiries can be directed to the corresponding authors.

## Ethics statement

The studies involving human participants were reviewed and approved by Nanjing first hospital. The patients/participants provided their written informed consent to participate in this study.

## Author contributions

ZJ, ZZ, and SW conceived, organized and supervised the project, and proofread the manuscript. ZJ, XQ and ZW collected and analyzed the data, and drafted the manuscript. XQ supervised statistical analysis. ZJ, YD and YP completed *H. pylori* laboratory identification and antimicrobial sensitivity testing. All authors contributed to the article and approved the submitted version.

## Fundings

This study was supported by grants from Special Funds for Key Research and Development Plans of Jiangsu Province in 2019 (NO. BE2019614), Nanjing health science and technology development special fund project plan (YKK20108) and Xinghuo Talent Program of Nanjing First Hospital.

## Conflict of interest

The authors declare that the research was conducted in the absence of any commercial or financial relationships that could be construed as a potential conflict of interest.

## Publisher’s note

All claims expressed in this article are solely those of the authors and do not necessarily represent those of their affiliated organizations, or those of the publisher, the editors and the reviewers. Any product that may be evaluated in this article, or claim that may be made by its manufacturer, is not guaranteed or endorsed by the publisher.
