# Supplementary material for: Antibiotic resistance of Helicobacter pylori isolated from patients in Nanjing, China: A cross-section study from 2018 to 2021
Source: Front Cell Infect Microbiol. 2022 Sep 8;12:970630. doi: 10.3389/fcimb.2022.970630 (PMC9493266; doi:10.3389/fcimb.2022.970630)
Supplement: Supplementary file 1 [file Table_1.docx]

**Supplementary Table 1.** Primers and probe for ARMS-PCR detection of the *H. pylori* 23S rRNA gene

| **Name** | **Sequence** |
| --- | --- |
| A2142A forward primer | 5’‐CTACCCGCGGCAAGACTGA‐3’ |
| A2142G forward primer | 5’‐CTACCCGCGGCAAGACTGG‐3’ |
| A2142C forward primer | 5’‐CTACCCGCGGCAAGACTGC‐3’ |
| A2143G forward primer | 5’‐CTACCCGCGGCAAGACGTAG‐3’ |
| Common reverse primer | 5’‐ATAGGTGGGAGGCTTTGAAGTA‐3’ |
| Common probe | 5’‐GACCCCGTGGACCTTTACTACAACT‐3’ |
